# Association Between Alcohol Consumption and the Risk of Barrett's Esophagus: A Meta-Analysis of Observational Studies

**DOI:** 10.1097/MD.0000000000001244

**Published:** 2015-08-14

**Authors:** Qin Xu, Wei Guo, Xingang Shi, Wei Zhang, Tianyi Zhang, Cheng Wu, Jian Lu, Rui Wang, Yanfang Zhao, Xiuqiang Ma, Jia He

**Affiliations:** From the Department of Health Statistics (QX, WG, TZ, CW, JL, RW, YZ, XM, JH), Second Military Medical University; Department of Gastroenterology (XS), Changhai Hospital, Second Military Medical University; and Department of Heath Services Management (WZ), Second Military Medical University, Shanghai, China.

## Abstract

The association between alcohol consumption and Barrett's esophagus (BE) remained uncertain and controversial in the previous studies. We performed a meta-analysis of observational studies to clarify the association.

We searched PubMed, Web of Science, and Embase for studies on alcohol consumption and risk of BE published before February 2015. A total of 20 studies reporting the association between alcohol consumption and the risk of BE were identified. Subgroup analyses, meta-regression analyses, sensitivity analyses, and publication bias tests were also performed. Several results from individual studies were pooled using a dose–response meta-analysis.

A total of 20 studies involving 45,181 participants and 4432 patients of BE were included in the meta-analysis. No association was found between alcohol consumption and BE (relative risk [RR] = 1.10, 95% confidence interval [CI] 0.96–1.27, I^2^ = 48.60%) in our study. In subgroup analysis, alcohol consumption was associated with an increased risk of BE in men (RR = 1.35, 95% CI 1.13–1.61, I^2^ = 0.00%) and Asian population (RR = 1.60, 95% CI 1.03–2.49, I^2^ = 60.60%). In beverage-specific consumption analysis, liquor was associated with an increased risk of BE (RR = 1.16, 95% CI 1.02–1.32, I^2^ = 0.00%). Multivariate meta-regression analysis suggested that geographic area, and adjusted age, sex, body mass index, and smoke, might explain 70.75% of the heterogeneity between the studies. We also found the inverse association (RR = 0.84, 95% CI 0.72–0.98, I^2^ = 0.00%) between alcohol consumption and BE among subjects when compared with population controls.

Overall, there was no significant association between alcohol consumption and BE. Alcohol consumption may be a risk factor of BE in men and Asian population, and liquor consumption may also increase the risk of BE. Significant inverse association was observed between alcohol consumption and BE, for comparisons with population controls.

## Abstract 2

Supplemental Digital Content is available in the text

## INTRODUCTION

Esophageal adenocarcinoma (EAC) has shown to be one of the most rapidly rising incidence of all malignancies in the Western world over the past decades.^1^ The incidence of Barrett's esophagus (BE), the premalignant precursor lesion of EAC, is also rising.^2,3^ The American Gastroenterological Association defines BE as a condition in which any extent of metaplastic columnar epithelium that predisposes to cancer development replaces the stratified squamous epithelium that normally lines the distal esophagus.^4^ BE was initially categorized as long segment (currently define as >3 cm) and short segment (currently define as ≤3 cm).^5^ BE affects 1% to 2% of the general population,^6^ and is the only known precancerous lesion for EAC.^7,8^ Compared with the general population, BE could increase the risk of developing EAC by 10 to 55 fold.^7–10^ Considering BE and its underlying condition is the major risk factor for EAC,^11,12^ understanding the causes of BE is a necessary step toward preventing EAC.

Important risk factors for BE include gastroesophageal reflux disease (GERD) symptoms, abdominal obesity, tobacco use, and male sex.^13^ However, it remains unclear whether alcohol consumption is truly associated with the present of BE, and whether patients’ drinking history could increase the risk stratification for BE. Previous studies have showed a weak association between alcohol drinking and EAC.^14–16^ However, recent studies of beverage-specific alcohol consumption also reported lower risk of BE and EAC associated with modest wine drinking,^17–20^ whereas others reported higher risk associated with total alcohol^9^ and liquor consumption.^18,21^ It is unclear whether these disparate results are due to measurement error in the assessment of alcohol consumption, or methodological differences in exposure definitions, or differences between the study populations, or effect modification by known causal factors for BE, or other aspects of the study design or analysis.

To date, no meta-analysis of the relationship between alcohol drinking and BE has been performed. With the aim to evaluate the effect of alcohol on the risk of BE, we therefore conducted a comprehensive meta-analysis of published case-control and cohort studies.

## METHODS

### Data Sources, Search Strategy, and Selection Criteria

This review was performed according to the Meta-analysis of Observational Studies in Epidemiology (MOOSE) guidelines.^22^

We carried out a literature search using the terms “Barrett's esophagus” or “Barrett's epithelium” or “Barrett syndrome” with “ethanol” or “alcohol” or “alcoholic beverages” to search PubMed, Embase and Web of Science databases for identification of articles published from 1976 to March 31, 2015. We also conducted manual searches of the reference lists of all the relevant original and review articles to identify additional eligible studies. A search for unpublished literature was not performed and authors were not contacted for missing data. Studies were included if they met the following inclusion criteria: studies used a case-control, nested case-control, or cohort study design; BE was diagnosed by the histologic finding of intestinal metaplasia within an endoscopic identified columnar-lined esophagus; and the risk point estimate was reported as relative risk [RR] or odds ratio [OR] and the corresponding 95% confidence intervals (CIs), or sufficient information provided to calculate these estimates. We excluded studies that did not meet the inclusion criteria. Specifically, studies were excluded for the following reasons: studies looked at endoscopic suspected BE patients; studies were present as proceedings and were not published as original articles. The literature search and inclusion or exclusion was independently undertaken by 2 investigators (QX and WG) using a standardized approach. Any inconsistencies between these 2 investigators were settled by the third investigator (XS) until a consensus was reached. Institutional review board approval and patient consent were not required for this meta-analysis of observational studies.

### Data Extraction and Quality Assessment

We performed the data extraction via a standardized data extraction form, collecting information on the author publication year, study location, study design, source of study population, sample size, assessment of alcohol consumption, age of subjects, proportion of males, follow-up time, the number of cases/noncases or person-year data, type of controls, effect estimate and its corresponding 95% CIs, and covariates adjusted in the statistical analysis. Quality assessment of each selected study was conducted by 2 investigators (QX and WG) using the Newcastle–Ottawa Scale (NOS).^23^ The NOS uses 2 different tools for case-control and cohort studies, and consists of 3 parameters of quality: selection, comparability, and exposure/outcome assessment. The NOS has developed a “star system” (range, 0–9) for the assessment of a maximum of 4 points for selection. A total score of 7 or greater was used to indicate high-quality studies, and a total score of 6 or lower indicated low-quality studies.

### Statistical Analysis

We examined the relationship between alcohol consumption and risk of BE on the basis of the RRs and 95% CIs (estimated by the OR and its 95% CIs in case-control and the hazard ratio and its 95% CIs in cohort studies) reported in each study. We used adjusted risk estimates whenever it is available; otherwise, we utilized or computed the unadjusted RRs. Because different measurement units were used to express alcohol consumption, we converted alcohol consumption levels into grams of ethanol per day for which details are available online (see supplementary table http://links.lww.com/MD/A372). We used fixed-effect models to evaluate the pooled RR with its 95% CI if there was no evidence of heterogeneity; otherwise, we used random-effect model.^24,25^ Next, we conducted a dose–response analysis in order to take into account the correlation between the log of RRs across categories of alcohol consumption for which details of the methods used have been described by Orsini.^26,27^ Only studies that reported RRs with their corresponding 95% CIs, for at least 3 quantitative categories were included. We examined a potential nonlinear dose–response relationship between alcohol consumption and BE among those studies reporting level-specific RR estimates with random-effects models. The *P* value for nonlinearity was calculated by testing the null hypothesis that the coefficient of the second spline was equal to zero. To investigate the sources of heterogeneity between the results of different studies, we carried out the following tests: heterogeneity tests, subgroup analysis, meta-regression analysis, and sensitivity analysis.^28,29^ The Cochran *Q* test and I^2^ statistic were used to explore the heterogeneity among studies.^30^ We considered *P* value was <0.10, and I^2^ value was >50% significantly statistical heterogeneity.^31^ Finally, by using the same methodology as for the subgroup analysis, we conducted stratified analyses by categories of sex, beverage type, geographic area, control type, alcohol consumption level, NOS score, adjusted age, adjusted sex, adjusted body mass index (BMI), and adjusted smoke to assess potential effect modification. Univariate meta-regression analysis was conducted first, after which the variables that were significant at the 0.1 level were entered into the multivariable model. To identify potentially influential studies, sensitivity analysis was also performed to examine whether the effect estimate was robust by repeating the random-effects meta-analysis after omitting 1 study at a time.

Publication bias was assessed by the Egger regression test and Begg test together with the visual inspection of the funnel plot.^32,33^ We also performed a sensitivity analysis by removing a specific study from the pooled analysis. All statistical analyses were carried out using Stata V.12.0 software (Stata, College Station, TX). A 2-tailed *P* value <0.05 was considered statistically significant.

## RESULTS

### Search Results and Study Characteristics

**FIGURE 1:**
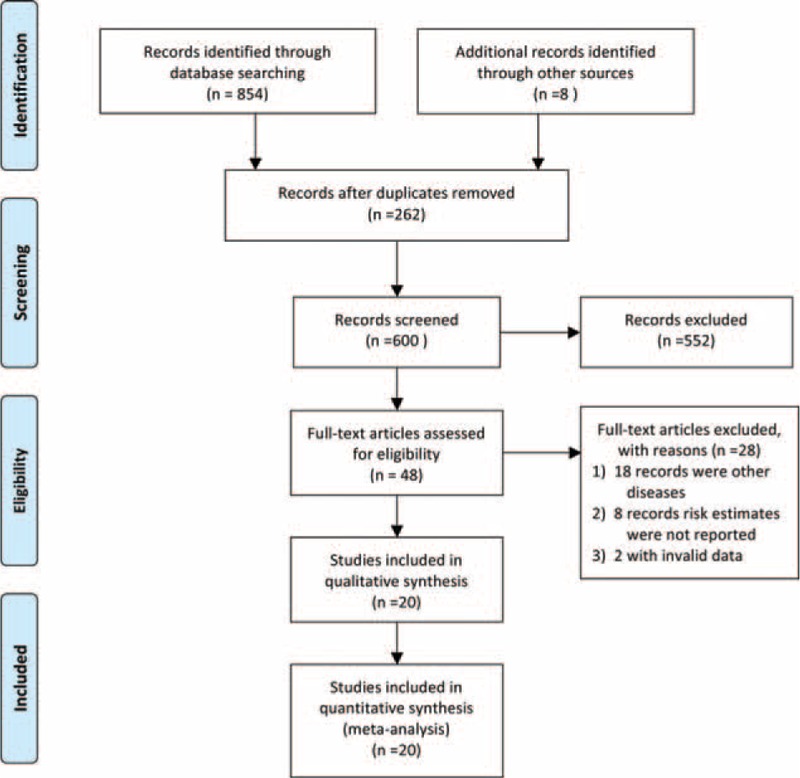
PRISMA flow diagram.

The study selection process is shown in Figure 1. A total of 862 articles were retrieved using the search strategy described, of which 814 were excluded according to the inclusion criteria, remaining 48 articles for further evaluation by full texts. One article published in Korean, which did not report the risk estimate, was excluded.^34^ Finally, 20 studies involving 45,181 participants and 4432 patients of BE were included in the meta-analysis after detailed evaluations. Among 20 studies, 12 case-control studies,^18,19,35–44^ 8 cohort studies,^9,21,45–50^ and 6 studies reporting categories of alcohol consumption were included to conduct the dose–response analysis of the relationship between liquor consumption and the risk of BE.^18,19,21,37,39,49^ Five records from 4 studies were included to conduct the dose–response analysis of the relationship between total alcohol consumption and the risk of BE for comparisons with population-based controls.^18,19,37,49^ The general characteristics of the included studies are shown in Table 1.

**TABLE 1:**
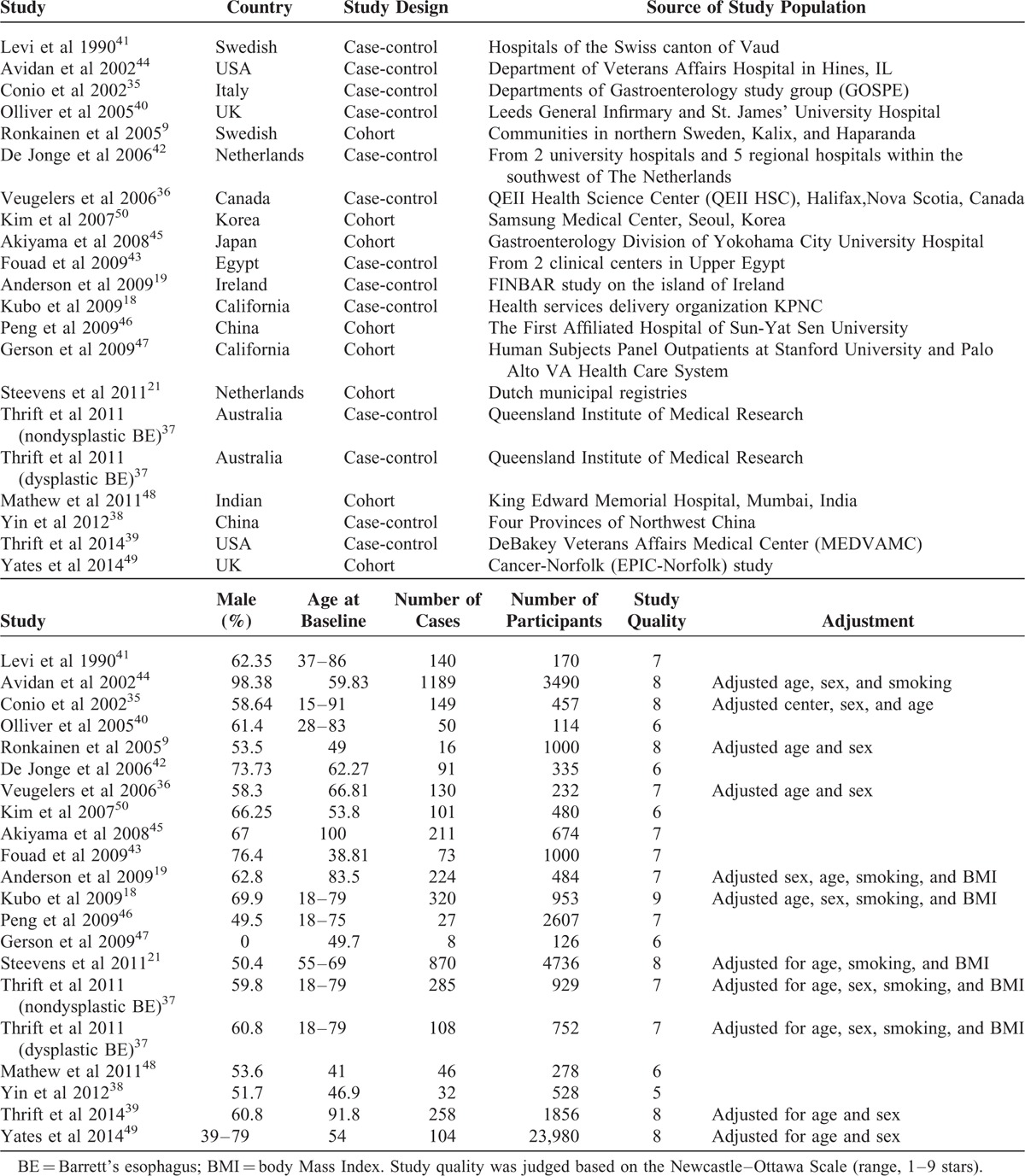
Characteristic of the Included Studies With Regard to Alcohol Consumption and Risk of Barrett's Esophagus

### Effects of Alcohol Consumption on BE

**FIGURE 2:**
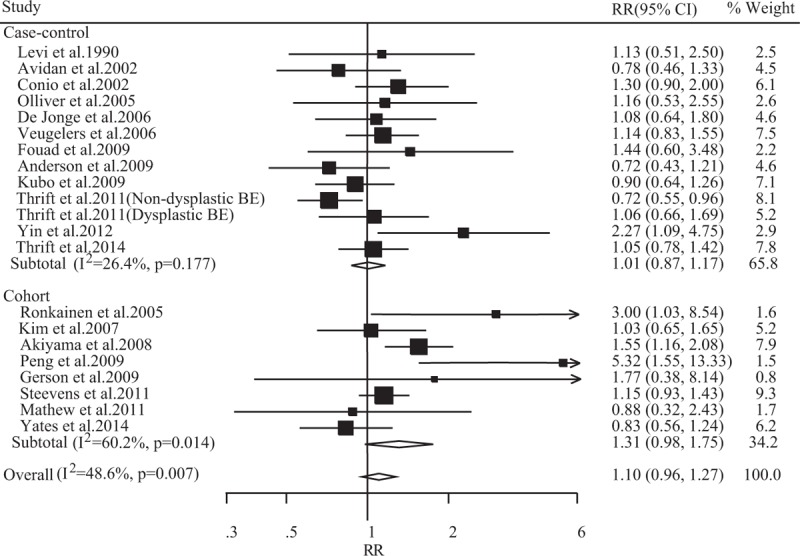
Summary relative risks (RRs) of Barrett's esophagus for alcohol consumption versus no alcohol consumption.

Figure 2 shows the forest plots of alcohol consumption and BE. The summary RR was 1.10 (95% CI 0.96–1.27), with heterogeneity (*P* = 0.007, I^2^ = 48.60%) and no publication bias was found (Egger test *P* = 0.169, Figure 4). The corresponding estimate of RRs was 1.01 (95% CI 0.87–1.17) for case-control studies, with heterogeneity (*P* = 0.177, I^2^ = 26.4%) and 1.31 (95% CI 0.98–1.75) for cohort studies, with heterogeneity (*P* = 0.014, I^2^ = 60.20%), respectively.

**FIGURE 3:**
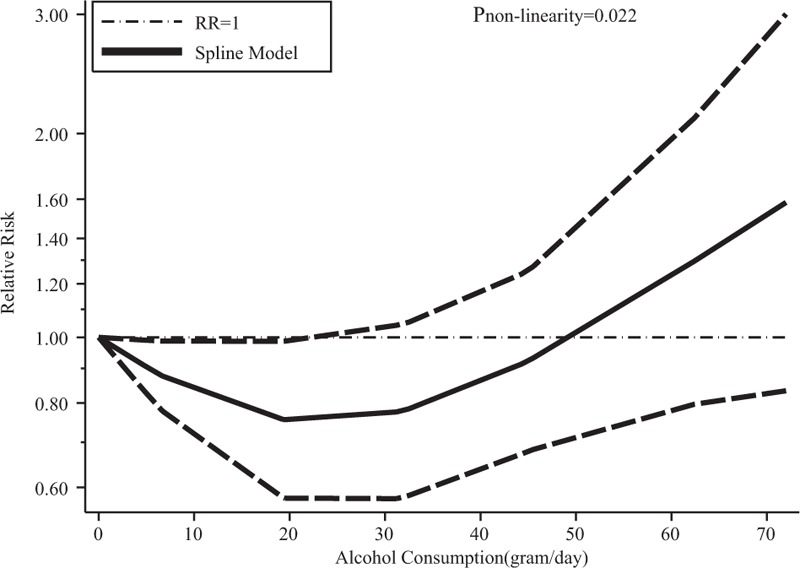
Dose–response relationship between alcohol consumption and risk of Barrett's esophagus for comparisons with population-based controls.

**FIGURE 4:**
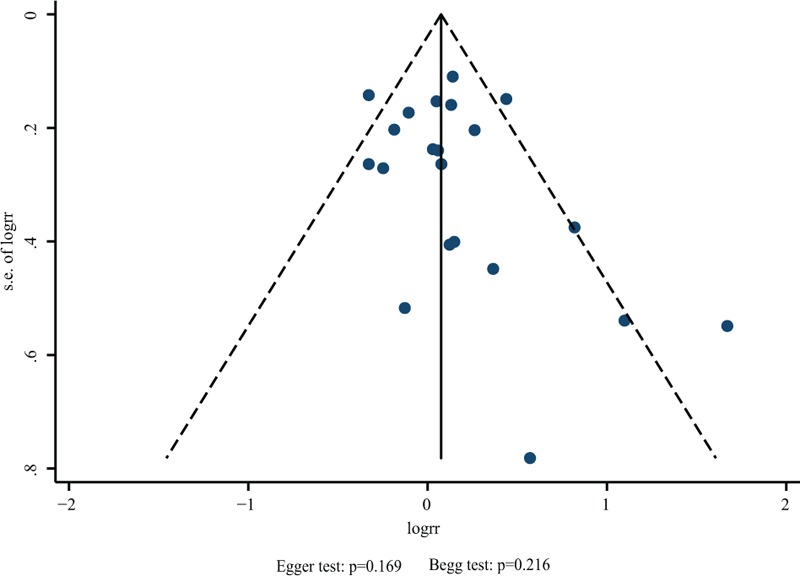
Funnel plot of log relative risk versus standard error of log relative risks.

### Subgroup Analysis

Furthermore, we conducted subgroup analysis to minimize heterogeneity among the included studies. In beverage-specific consumption analysis, liquor was associated with an increased risk of BE (RR = 1.16, 95% CI 1.02–1.32, I^2^ = 0.00%). The dose–response meta-analysis did not show evidence of a nonlinear relationship between alcohol and risk of BE (*P* = 0.632). Also, no linear relationship was observed (RR = 1.05, 95% CI 0.99–1.11) for every 5 g/d increase in alcohol. We failed to reveal consistent associations between beer, wine, spirits, and the risk of BE. Nevertheless, we found that there was an inverse association (RR = 0.84, 95% CI 0.72–0.98, I^2^ = 0.00%) for BE among subjects with GERD when compared with population controls in 6 records from 5 studies,^18,19,37,49,50^ which indicated that there might be a U-shaped nonlinear trend between alcohol consumption and risk of BE (*P*_nonlinearity_ = 0.022, Figure 3). The dose–response analysis suggested that an alcohol consumption of <23 g/d might have a potential beneficial effect on BE compared with population control. Alcohol consumption was not associated with the risk of BE when compared with hospital controls and GERD controls (Table 2).

**TABLE 2:**
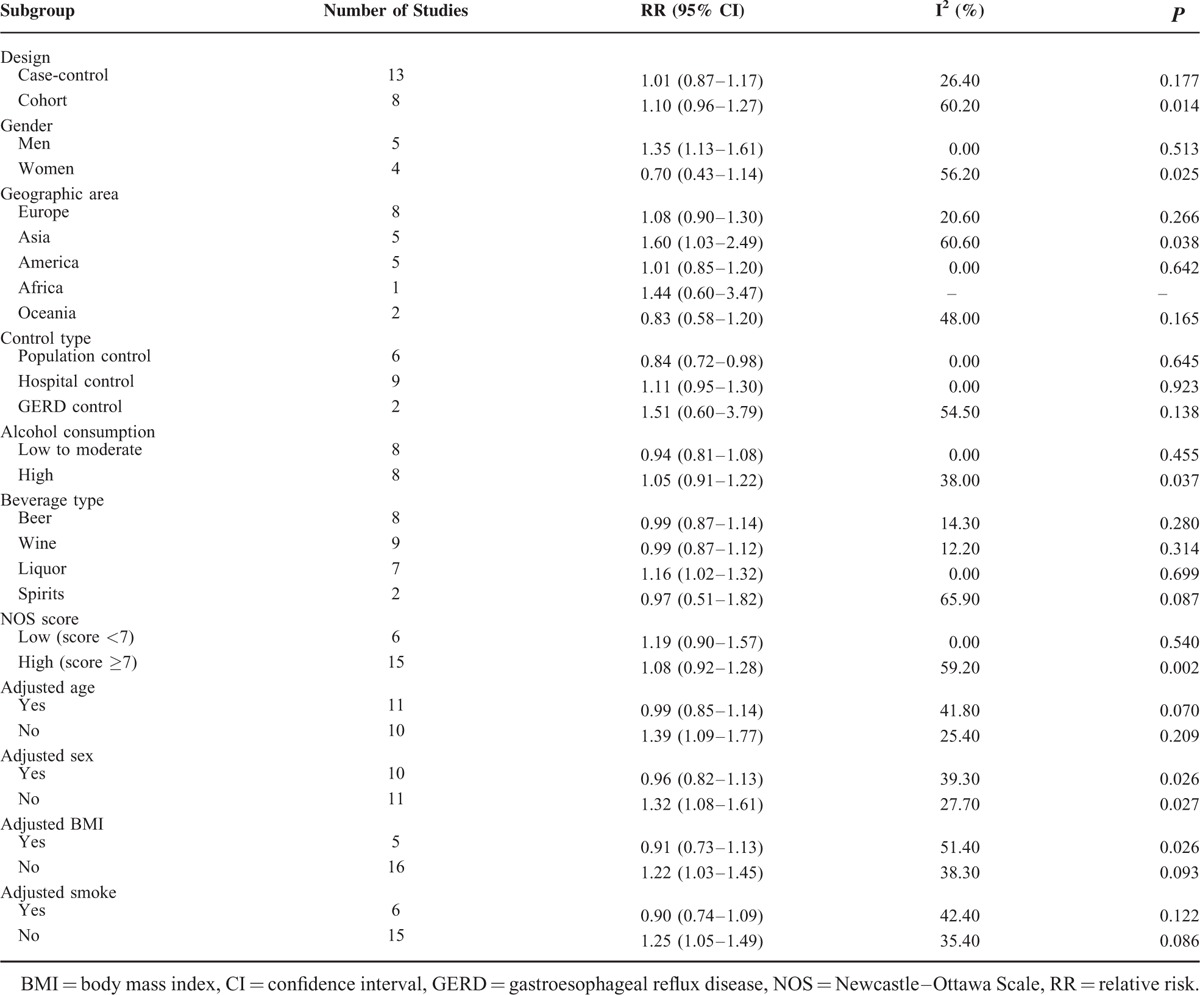
Subgroup Analysis of Barrett's Esophagus for Alcohol Consumption Versus No Alcohol Consumption

Alcohol consumption was associated with an increased risk of BE in men (RR = 1.35, 95% CI 1.13–1.61, I^2^ = 0.00%) and Asian population (RR = 1.60, 95% CI 1.03–2.49, I^2^ = 60.60%). We evaluated whether adjusted age, sex, BMI, and smoke modified the association between alcohol consumption and the risk of BE (Table 2). There were statistically significant increased risk of alcohol consumption on the incidence of BE with unadjusted age (RR = 1.39, 95% CI 1.09–1.77, I^2^ = 25.40%), unadjusted sex (RR = 1.32, 95% CI 1.08–1.61, I^2^ = 27.70%), unadjusted BMI (RR = 1.22, 95% CI 1.03–1.45, I^2^ = 38.30%), and unadjusted smoke (RR = 1.25, 95% CI 1.05–1.49, I^2^ = 35.40%).

### Meta-Regression

We used publication year, study design, study quality, total participants, male, geographic area, adjusted age, adjusted sex, adjusted BMI, and adjusted smoke as explanatory covariates. Univariate meta-regression analysis was performed first. Results of the univariate analysis are shown in Table 3. In univariate meta-regression analysis, the regression coefficients of geographic area in Asia (*P* = 0.009), adjusted age (*P* = 0.027), adjusted sex (*P* = 0.025), adjusted BMI (*P* = 0.066), and adjusted smoke (*P* = 0.026) were significant at the level of 0.1. Thus, the above 5 covariates were entered into the multivariate meta-regression analysis whose results are shown in Table 4. The τ^2^ changed from 0.0456 to 0.01334 after including these 5 covariates in the model, which means that 70.75% of heterogeneity between the studies can be explained by these covariates.

**TABLE 3:**
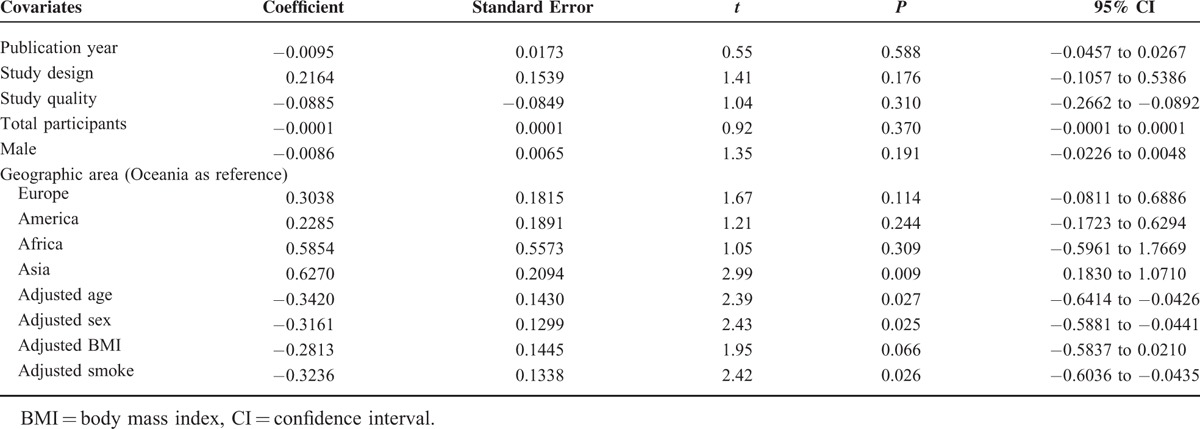
Univariate Meta-Regression Analysis for the Potential Variables Between Studies

**TABLE 4:**
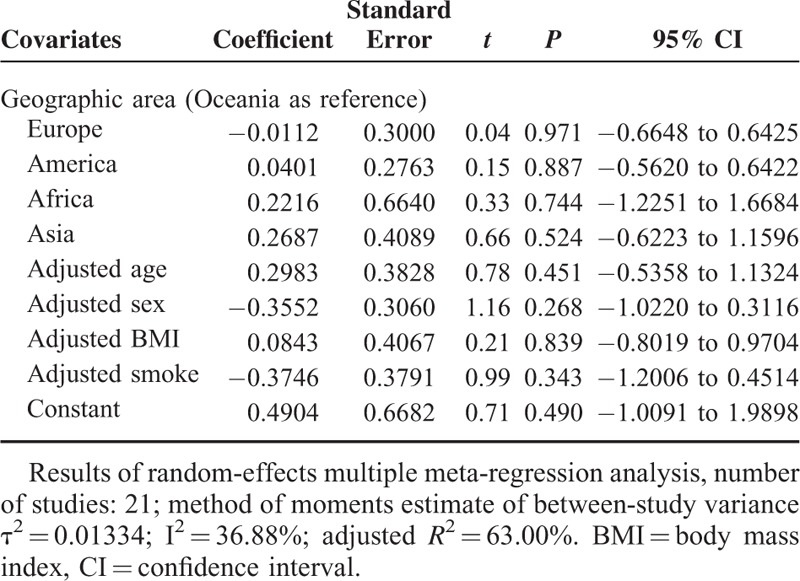
Multivariate Meta-Regression Analysis for the Potential Variables Between Studies

### Sensitivity Analysis

The results of the sensitivity analysis in Table 5 indicated that the conclusion was not affected by sequential exclusion of any studies except 1 study of nondysplastic BE.^37^ The total result was completely different when we excluded this record (RR = 1.13, 95% CI 1.03–1.25, I^2^ = 36.40%, *P* = 0.053).

**TABLE 5:**
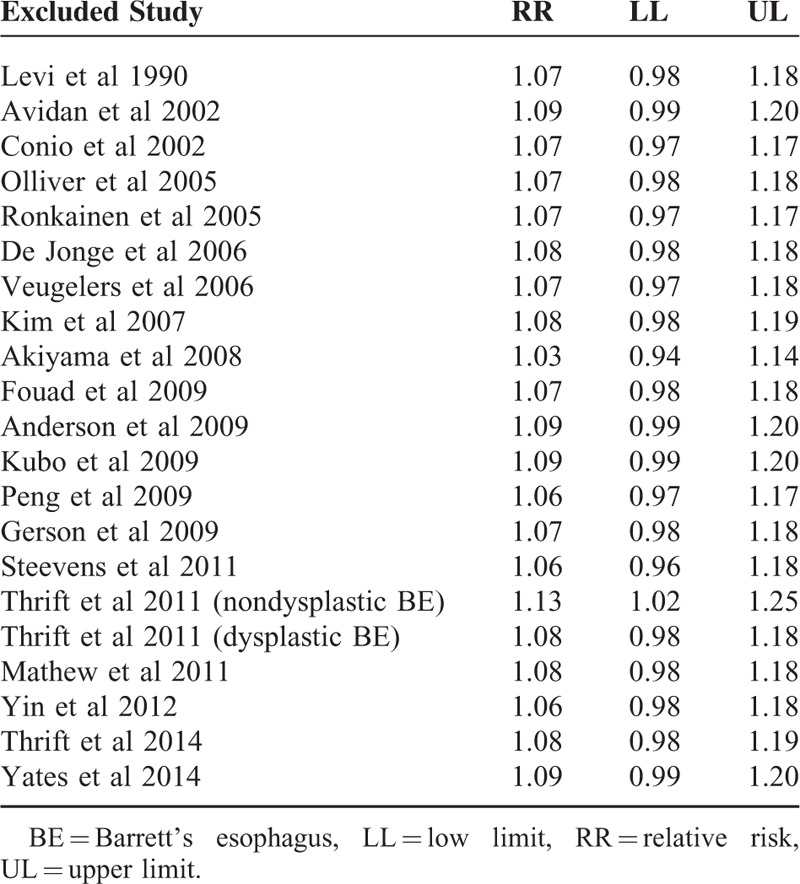
Sensitivity Analysis

## DISCUSSION

Our meta-analysis identified 20 observational studies through a broad search of manually reviewed databases and rigorous inclusion criteria. Findings from this study showed that total alcohol consumption was not a risk factor for BE. In subgroup analysis, alcohol consumption was associated with an increased risk of BE in men and Asian population. We found that alcohol was a risk factor for BE among subjects with GERD by comparing with GERD controls who lack BE on endoscopy. However, compared with population controls, there was an inverse association between alcohol consumption and BE. In beverage type analysis for total alcohol consumption, liquor was associated with an increased risk of BE. The association between alcohol consumption and BE was also modified by other factors, including age, sex, BMI, and smoke.

Studies have indicated that male sex might increase the risk of BE,^38,51,52^ which was confirmed by the present study. Our study also found that the risk of BE increased with increasing alcohol consumption in Asian population, which is in accordance with the results of previous studies in Japan and Korea.^34,45^ However, the relationship between alcohol consumption and BE was not found in Westerns.^20,53^ This might be due to the different disease pattern of BE between Asians and Westerns because most BE patients in Asia are the short-segment type.^54^ It is not difficult to find that none of the Asian studies included in our meta-analysis had adjusted estimates. Thus, the results that alcohol consumption was associated with increased risk of BE among Asians are possibly due to some potentially confounding factors, which need to be further explored.

Subgroup analysis indicated that there was a statistically significant inverse association for BE among subjects with GERD when compared with population-based controls. A large population-based case-control study conducted by Thrift et al^37^ found that compared with population controls, these lifelong nondrinkers and consumption of <41 drinks/wk of total alcohol consumption throughout the life were less likely to have nondysplastic BE. Thrift's another pooled analysis showed that compared with population-based controls, there was a borderline statistically significant inverse association between any alcohol consumption and the incidence of BE.^20^ A possible explanation for these somewhat discrepant findings might be that most BE patients drink more alcohol in early life, and then slowly reduce the intake as a result of either their discomfort symptoms or diagnosis.

The association between liquor consumption and BE was first identified by Ritenbaugh.^55^ Veugelers^36^ also reported that increased liquor consumption was a risk factor for both GERD and BE. There are several potential mechanisms through which different alcohol type may be associated with BE. First, liquor drinkers are less likely to consume their alcohol beverage with food. Consumption of alcohol without food may directly damage the lining of the esophagus and increase the esophagitis process, whereas mixed liquor consumption cannot increase the risk.^56^ Another possibility is that liquor consumption is proxy for some unmeasured unhealthy lifestyle, such as eating fewer fruits and vegetables and having high BMI, which in turn explain the significant risk associations, because many studies have reported that frequency of general alcohol consumption and type of beverage are related to many factors.^57,58^

Sensitivity analysis indicates that the association between alcohol consumption and BE is completely different by exclusion of nondysplastic BE study.^37^ Thrift's study found that there was evidence of an inverse trend for nondysplastic BE, nondysplastic BE patients reported lower intakes than population controls, the possibility seemingly protective effect of lifetime alcohol consumption, as BE patients may refrain from alcohol consumption over time after enduring prolonged reflux discomfort.^37^ Therefore, whether alcohol consumption increased risk of progression of nondysplastic BE to high-grade dysplasia/adenocarcinoma or not need to be further explored.

Several strengths of the current study should be highlighted. The main strength is that it is the first meta-analysis focusing on the association between alcohol consumption and the incidence of BE. Furthermore, the ascertainment of outcome is based on endoscopy and histological finding in all studies, and the majority of studies included evaluate multiple confounders such as age, sex, BMI, smoke, and so on.

There are also several potential limitations to the study. First, limited by the observational design, exclusion of potential confounders from other BE risk factors cannot be ruled out. A meta-analysis is not able to address problems with confounding factors that could be inherent in the original studies. However, in most studies included in this meta-analysis, the investigators had adjusted for major potential confounders, including sex, age, BMI, and smoke. Marked heterogeneity is also observed across these studies which may reflect differences in study design, study population, and adjustment for confounders. Nevertheless, we carried out stratified analysis, meta-regression, and sensitivity analysis to explore this potential bias. Another limitation is the different definition of alcohol consumption among studies, which might result in heterogeneity in our meta-analysis. Some studies used the grams of alcohol to weigh the alcohol consumption, whereas others used drinks of alcohol. We converted all measures into grams alcohol per day using the definitions that reported in the studies.

## CONCLUSIONS

In summary, the results of this study suggested that there is no association between total alcohol consumption and BE risk. However, alcohol consumption was associated with an increased risk of BE in men and Asians. In beverage analysis, liquor consumption was associated with an increased risk of BE either. We found that alcohol was a risk factor for BE in GERD patients. However, when compared with population controls, there was an inverse association. The dose–response meta-analysis suggested that there might be a U-shaped nonlinear trend between alcohol consumption and risk of BE, and an alcohol consumption of <23 g/d might have a potential beneficial effect on BE.
